# What are the outcomes of core decompression in patients with avascular necrosis? Protocol for a systematic review

**DOI:** 10.12688/f1000research.22167.1

**Published:** 2020-01-31

**Authors:** Octavian Andronic, Haitham Shoman, Ori Weiss, Vikas Khanduja

**Affiliations:** 1 Orthopaedic Surgery, Balgrist University Hospital, University of Zürich, Zürich, 8008, Switzerland; 2 Young Adult Hip Service, Department of Trauma and Orthopaedics, Addenbrooke's - Cambridge University Hospital, Cambridge, CB2 0QQ, UK; 3 Department of Global Health and Social Medicine, Harvard Medical School, Boston, Massachusetts, 02115, USA; 4 Department of Orthopaedic Surgery, Meir Medical Center, Kfar-Saba, 4428164, Israel

**Keywords:** Core decompression, hip, avascular necrosis, hip preservation, femoral head, avn.

## Abstract

**Background:** Core decompression is a hip preserving surgical procedure that is used to treat avascular necrosis (AVN) of the femoral head. The eventual clinical and radiological outcome following this procedure is varied in literature. Also, the time to a total hip replacement (THR) from the index procedure and the percentage of patients subsequently undergoing a THR is controversial. Furthermore, there are multiple surgical methods along with multiple augmentation techniques and various classification and staging systems described. The purpose of this systematic review, therefore, is to analyse the outcomes following decompression only, excluding any augmentation techniques for non-traumatic AVN of the femoral head.

**Methods: **This protocol is being developed in line with the PRISMA-P guidelines. The search strategy includes articles from Medline, Embase, Google Scholar, CINHAL and Cochrane library. The review and screening will be done by two independent reviewers. Review articles, editorials and correspondences will be excluded. Articles including patients with sickle cell disease and with core decompression where augmentation is used will be excluded. The risk of bias and quality of articles will be assessed using the Joanna Briggs Institute Critical Appraisal Checklist for the different study designs included.

**Discussion: **This study will be a comprehensive review on all published articles having patients with AVN of the femoral head and undergoing core decompression surgery only. The systematic review will then define the outcomes of the core decompression surgery based on clinical and radiological outcomes. Each outcome will include the different stages within it and finally, the total mean time to THR will be calculated. This will then be followed by assessing the cumulative confidence in evidence from all the data collected using the GRADE tool.

**Registration:** This systematic review is registered in the International Prospective Register for Systematic Reviews and Meta-analysis (PROSPERO) under the registration number: CRD42018100596

## Introduction

Osteonecrosis or avascular necrosis (AVN) of the femoral head is a challenging condition that eventually leads, in the majority of cases, to a total hip replacement (THR) ^1– 3^. The patients affected by the condition are usually young and therefore may require revision surgery and multiple further procedures ^4^. The aetiology is varied and includes multiple conditions that can lead to a reduced blood supply in the femoral head: oral corticosteroids, systemic lupus erythematosus, binge consumption of alcohol, Gaucher disease, sickle cell anaemia, trauma, thrombosis amongst others ^5^. Furthermore, staging systems for AVN are different across the literature and pose a significant problem in assessing surgical indications and stratifying outcome ^6^. The most common classification systems used are: Ficat ^7^/ Modified Ficat ^8^; “University of Pennsylvania”/Steinberg ^6^; and ARCO (Association Research Circulation Osseous) ^9^.

Core decompression is a common surgical procedure that has been used earlier on in the disease process to decrease the intraosseous pressure in the femoral head, relieve pain and potentially re-establish blood flow. Furthermore, multiple augmentation techniques have recently been described that seem to significantly improve the outcome following this procedure ^10, 11^.

However, the eventual outcome and time to THR following this procedure remains controversial ^12– 16^. It is also not clear whether a mechanical decompression alone is sufficient and efficient in all stages of AVN in order to prevent progression and delay the need of a THR. To the best of our knowledge, the largest published systematic review on this subject included only four studies for analysis ^17^.

The purpose of this study, therefore, was to assess the outcomes of core decompression without any augmentation for nontraumatic AVN of the femoral head.

## Methods/design

### Study design

The Preferred Reporting Items for Systematic Reviews and Meta-analysis – Protocol (PRISMA-P) guidelines will be used to develop the protocol of the study. The manuscript will then be developed using the PRISMA statement and flowchart. This systematic review is registered in the International Prospective Register for Systematic Reviews and Meta-analysis (PROSPERO) under the registration number: CRD42018100596.

### Search strategy

The search for articles will include several databases, Medline, Embase, Google Scholar and the Cochrane library. There will be no restriction on dates and articles will be included from inception. The search strings will include articles looking at patients with AVN and having core decompression and they will then be combined using the Boolean terms AND/OR. A total of eight combinations of the following keywords will be used: “femoral head” with “osteonecrosis”, “avascular necrosis”, “aseptic necrosis”, “avn” with the terms - “core decompression” or “surgery”.

### Study selection

First, a blinded and independent process of selection based on title and abstract will be made by two authors (OA and HS). Secondly, a thorough analysis of eligible studies was performed by evaluating full texts. Studies will then be screened according to the inclusion and exclusion criteria ( Table 1). Articles included would be in English language, looking at those suffering from AVN and those who had core decompression only. Reviews, editorials and commentaries will be excluded. The PICO tool is used to formulate the research question. The participants will be everyone with no restriction to age, race or gender, the intervention is core decompression, there will be no comparator, and the outcomes will include clinical and radiological ( Table 2).

**Table 1.** Inclusion and exclusion Criteria

Inclusion criteria	Exclusion criteria
• Studies that included core decompression alone as the only intervention • Studies on patients with avascular necrosis • Case reports, case series, case control, cohort studies and randomized controlled trials	• Non-English articles • Any review/hypothesis/technique articles, editorials, commentaries and non-clinical articles • Patients with core decompression in combination with another surgery or augmentation technique.

**Table 2.** PICO tool

Population	All races and genders with no age or geographical restrictions
Intervention	Core decompression
Comparators	Not applicable
Outcomes	Clinical and radiological outcomes and time to total hip replacement

### Data extraction

The selected articles will then be exported to Mendeley reference manager software and all duplicates will be removed electronically and manually. The final number of included articles will then be assessed for full text review and data will be extracted based on a pre-determined set of variables. Two reviewers (AO and OW) will assess and screen and if there is any discrepancy, a third more senior reviewer (VK) will be invited to advice until consensus is met between all authors. Data extracted will then be pulled into a spreadsheet with the pre-determined variables on Microsoft Excel v16.21.

The extracted data will based on the following table headings: author, study setting (country and year), number of included hips, average follow up, gender percentages, average age, mean Body Mass Index (BMI), preliminary diagnosis (primary etiology), stage of disease, surgical technique, clinical outcome (with preoperative and postoperative results where applicable), radiological outcome and time to THR.

### Data analysis and synthesis

The risk of bias and quality of studies will be evaluated using the Joanna Briggs Institute Critical Appraisal Checklist ^18^ for each study design due to its rigor in assessing the methodological integrity of studies. The outcomes of core decompression will include clinical and radiological outcomes. In addition, the total mean time for hip replacement among all the studies will be estimated. Based on the quality of studies included, a meta-analysis might be conducted across the studies if there was limited heterogeneity in the data.

### Amendments

In cases of changes in the existing protocol that significantly would affect the accuracy of data, scope of the investigation, or scientific quality of the study, edits will be performed and a newer version that would be in accordance with the final systematic review, will be provided and published.

### Dissemination

The systematic review is planned to be submitted upon completion to an orthopaedic peer-reviewed journal with global audience and then uploaded under copyright conditions to further dissemination platforms, such as Research Gate and others.

## Study status

Ongoing.

## Discussion

Classification systems, outcome measures and reporting systems are highly variable amongst studies assessing and reporting the outcome of core decompression for AVN of the femoral head. From distinct classifications (Ficat or its modification, Steinberg, ARCO) to varied clinical scores (Harris Hip Score/D’Aubigne/Visual Analogue Scale), all have been described and used in the literature.

Furthermore, the concept of “procedural success” is not absolute. Whilst most studies consider the absence of radiological progression of disease to be the main finding that suggests success, other authors interpret clinical improvement as success, even in the presence of radiological deterioration. Therefore, the heterogeneity of interpretation of success in the studies extracted may not allow for a uniform representation. As such, the results will be represented in separate categories, based on the classification system that was used and divided into radiological progression and another category of clinical improvement.

The strengths of our study will be represented by the largest patient pool and rigorous exclusion criteria that will be used. Any collateral influence of aetiology (traumatic), systemic disease (sickle cell crisis) or technique heterogeneity (presence of augmentation or bone grafts) will be excluded. Also, there will be a tenacious stratification based on stage of the disease even in the presence of a variety of classification systems. Ultimately, the following questions will be evaluated and answered:

1) Does core decompression accomplish postoperative pain relief/clinical or functional improvement?

2) In what percentage of the patients does core decompression achieve a cessation of radiological progression?

3) What percentage of patients undergoing core decompression without augmentation, ultimately require a THR?

4) What is the average time to requiring a THR following core decompression?

## Data availability

### Underlying data

No data is associated with this work.

### Reporting guidelines

Figshare: PRISMA-P checklist for ‘What are the outcomes of core decompression in patients with avascular necrosis? Protocol for a systematic review’, https://doi.org/10.6084/m9.figshare.11720289.v3 ^19^.

## Author information

VK (MD, MA, MSc, FRCS(Orth)) is a Consultant Orthopaedic Surgeon at Addenbrooke’s - Cambridge University Hospital, England, UK. VK is also an Associate Lecturer at the University of Cambridge and the Associate Editor at the Bone and Joint Journal. He is also the Chair of SICOT Education Academy and Chair of the UK’s non-arthroplasty hip registry. OA (MD) is VK’s research fellow and orthopaedic surgery resident at the Balgrist University Hospital in Zurich, Switzerland. OW (MD) is VK’s research fellow and orthopaedic surgery resident at the Meir Medical Center, Kfar-Saba, Israel. HS (MD, DIC, MPH) is also VK’s research fellow and a Global Surgery Research Fellow at Harvard Medical School, Boston, MA, USA.
