# Supplementary material for: What are the outcomes of core decompression in patients with avascular necrosis? Protocol for a systematic review
Source: F1000Res. 2020 Jan 31;9:71. [Version 1] doi: 10.12688/f1000research.22167.1 (PMC7101011; doi:10.12688/f1000research.22167.1)
Supplement: Peer review history

# Peer review: Reviewer response for version 1

**DOI:** 10.5256/f1000research.24446.r61725

**Published:** 2020-03-26

**Authors:** Andreas Roth

**Affiliations:** 1 Department of Orthopedic, Trauma and Plastic Surgery, University Clinic of Leipzig, Leipzig University, Leipzig, Germany

The aim of the preliminary study is to examine the results of core decompression in avascular necrosis as part of a systematic review. The authors have presented a complete study protocol for this. In principle, study design and methods are suitable to achieve the goal.

 Because of the heterogeneity of the present work, they want to evaluate the results in different categories, which are based on the respective classification system and differ in terms of radiological progression and the improvement of the clinical findings.

 The authors must be aware that the number of patients described is relatively small. I took the liberty to list the literature I know.

 In fact, the papers available differ with regard to the primary endpoint. But the classification systems are also different. Last but not least, the surgical techniques used are different, and sometimes not exactly specified. These are the potential limitations of this work.

 Ultimately, however, it is of great interest for all those who deal with this clinical picture to obtain such results. Therefore, I would like to support this work and advocate the indexing of the project.

I confirm that I have read this submission and believe that I have an appropriate level of expertise to confirm that it is of an acceptable scientific standard.

## References

[1] : [The value of core decompression in treatment of femur head necrosis]. *Orthopade*.2000;29(5) : 10.1007/s001320050463 420-9 10.1007/s001320050463 10875136

[2] : Core decompression and conservative treatment for avascular necrosis of the femoral head: a meta-analysis. *Am J Orthop (Belle Mead NJ)*.2000;29(3) :187-94 10746469

[3] : Osteonecrosis of the femoral head. A prospective randomized treatment protocol. *Clin Orthop Relat Res*.1991;140-51 2060201

[4] : Preventing collapse in early osteonecrosis of the femoral head. A randomised clinical trial of core decompression. *J Bone Joint Surg Br*.1995;77(6) :870-4 7593097

[5] : Core decompression for avascular necrosis of the femoral head: correlation between long-term results and preoperative MR staging. *Radiology*.1990;175(2) : 10.1148/radiology.175.2.2326478 533-6 10.1148/radiology.175.2.2326478 2326478

[6] : Survival analysis of hips treated with core decompression or vascularized fibular grafting because of avascular necrosis. *J Bone Joint Surg Am*.1998;80(9) : 10.2106/00004623-199809000-00004 1270-5 10.2106/00004623-199809000-00004 9759810

[7] : Evaluation of core decompression for early osteonecrosis of the femoral head. *Arch Orthop Trauma Surg*.2000;120(5-6) : 10.1007/s004020050456 241-4 10.1007/s004020050456 10853887

[8] : Core decompression in atraumatic osteonecrosis of the hip. *J Arthroplasty*.2000;15(2) : 10.1016/s0883-5403(00)90132-3 171-8 10.1016/s0883-5403(00)90132-3 10708081

[9] : Joint-preserving operations for idiopathic avascular necrosis of the femoral head. Results of core decompression, grafting and osteotomy. *J Bone Joint Surg Br*.1988;70(1) :78-84 327670310.1302/0301-620X.70B1.3276703

[10] : Electrical stimulation of osteonecrosis of the femoral head. *Semin Arthroplasty*.1991;2(3) :214-21 10149658

[11] : Core decompression versus nonoperative management for osteonecrosis of the hip. *Clin Orthop Relat Res*.1996; 10.1097/00003086-199603000-00020 169-78 10.1097/00003086-199603000-00020 8595753

[12] : Physical therapy alone compared with core decompression and physical therapy for femoral head osteonecrosis in sickle cell disease. Results of a multicenter study at a mean of three years after treatment. *J Bone Joint Surg Am*.2006;88(12) : 10.2106/JBJS.E.01454 2573-82 10.2106/JBJS.E.01454 17142406

[13] : The conservative treatment of osteonecrosis of the femoral head. A comparison of core decompression and pulsing electromagnetic fields. *Clin Orthop Relat Res*.1989;209-18 2582669

[14] : Treatment for osteonecrosis of the femoral head: comparison of extracorporeal shock waves with core decompression and bone-grafting. *J Bone Joint Surg Am*.2005;87(11) : 10.2106/JBJS.E.00174 2380-7 10.2106/JBJS.E.00174 16264111

[15] : Bone marrow edema syndrome of the femoral head: treatment with the prostacyclin analogue iloprost vs. core decompression: an MRI-controlled study. *Wien Klin Wochenschr*.2005;117(4) : 10.1007/s00508-005-321-3 130-5 10.1007/s00508-005-321-3 15847192

# Peer review: Reviewer response for version 1

**DOI:** 10.5256/f1000research.24446.r60053

**Published:** 2020-03-23

**Authors:** Tony Andrade

**Affiliations:** 1 Department of Trauma and Orthopaedics, Royal Berkshire Hospital, Royal Berkshire NHS Foundation Trust, Reading, UK

The authors have submitted a study protocol for a study in which they set out to determine the outcomes of core decompression (without any augmentation procedure) for non-traumatic avascular necrosis of the femoral head. They aim to carry out a systematic review in line with PRISMA-P guidelines, with the review and screening carried out by two independent reviewers.

 Their study design and the described methods are completely appropriate.The outcomes will be based on clinical and radiological outcomes, and the mean time to Total Hip Replacement will also be calculated. Finally the GRADE tool will be used to assess the cumulative confidence in evidence from all the data collected.

 Previous authors have concluded from their meta-analyses, which mostly employed augmentation treatments, that as they could not find any differences in the rates of THA conversion and radiologic progression across all core decompression modalities and non-operative treatment, their results question the assumption that core decompression changes the natural course of avascular necrosis of the femoral head. The authors need to consider this.

 One of the potential limitations with previous meta-analyses has been the relatively low numbers of cases, and so the authors of this study need to be able to aim for higher numbers in their review.

 Previous studies have concluded with a call for very large scale randomised controlled trials to confirm the effectiveness of core decompression in itself. This further systematic review may well add to this call.

 I have also added in two citations that the authors should review, as these further inform the subject.

 The study is a very valuable one that aims to inform on the outcomes of decompression alone (without augmentation techniques) for AVN of the femoral head. This is a very important topic in the hip preservation setting and these results will add significant value to the existing literature. I therefore fully support this study, with only the minor points I have detailed above.

I confirm that I have read this submission and believe that I have an appropriate level of expertise to confirm that it is of an acceptable scientific standard.

## References

[1] : Joint-preserving procedures for osteonecrosis of the femoral head. *EFORT Open Rev*.2019;4(12) : 10.1302/2058-5241.4.180073 647-658 10.1302/2058-5241.4.180073 32010453PMC6986391

[2] : The natural history of untreated asymptomatic osteonecrosis of the femoral head: a systematic literature review. *J Bone Joint Surg Am*.2010;92(12) : 10.2106/JBJS.I.00575 2165-70 10.2106/JBJS.I.00575 20844158
